# Naming fMRI-guided white matter language tract volumes influence naming decline after temporal lobe resection

**DOI:** 10.1007/s00415-024-12315-2

**Published:** 2024-04-07

**Authors:** Karin Trimmel, Sjoerd B. Vos, Lawrence Binding, Lorenzo Caciagli, Fenglai Xiao, Louis A. van Graan, Matthias J. Koepp, Pamela J. Thompson, John S. Duncan

**Affiliations:** 1 Department of Neurology, Medical University of Vienna, Vienna, Austria; 2 Epilepsy Society MRI Unit, Epilepsy Society, Chalfont St Peter, UK; 3 Department of Clinical and Experimental Epilepsy, UCL Institute of Neurology, London, UK; 4 Centre for Medical Image Computing, Department of Computer Science, University College London, London, UK; 5 Neuroradiological Academic Unit, UCL Queen Square Institute of Neurology, London, UK; 6 Centre for Microscopy Characterisation and Analysis, University of Western Australia, Nedlands, Australia; 7 Department of Neurology, Inselspital, Sleep-Wake-Epilepsy-Center, Bern University Hospital, University of Bern, Bern, Switzerland

**Keywords:** Language fMRI, Tractography, Language tracts, Temporal lobe epilepsy, Epilepsy surgery, Naming outcome

## Abstract

**Objective:**

The aim of this study was to explore the relation of language functional MRI (fMRI)-guided tractography with postsurgical naming decline in people with temporal lobe epilepsy (TLE).

**Methods:**

Twenty patients with unilateral TLE (9 left) were studied with auditory and picture naming functional MRI tasks. Activation maxima in the left posterobasal temporal lobe were used as seed regions for whole-brain fibre tractography. Clinical naming performance was assessed preoperatively, 4 months, and 12 months following temporal lobe resection. Volumes of white matter language tracts in both hemispheres as well as tract volume laterality indices were explored as moderators of postoperative naming decline using Pearson correlations and multiple linear regression with other clinical variables.

**Results:**

Larger volumes of white matter language tracts derived from auditory and picture naming maxima in the hemisphere of subsequent surgery as well as stronger lateralization of picture naming tract volumes to the side of surgery correlated with greater language decline, which was independent of fMRI lateralization status. Multiple regression for picture naming tract volumes was associated with a significant decline of naming function with 100% sensitivity and 93% specificity at both short-term and long-term follow-up.

**Interpretation:**

Naming fMRI-guided white matter language tract volumes relate to postoperative naming decline after temporal lobe resection in people with TLE. This can assist stratification of surgical outcome and minimize risk of postoperative language deficits in TLE.

**Supplementary Information:**

The online version contains supplementary material available at 10.1007/s00415-024-12315-2.

## Introduction

Anterior temporal lobe resection (ATLR) is an effective treatment option for medically refractory temporal lobe epilepsy (TLE) [1], but despite advancements in surgical procedures and neuroimaging over the past decades, postoperative decline of naming and word finding functions remains a major concern, affecting up to 25–50% of patients after dominant temporal lobe resections [2–6]. After non-dominant temporal lobe resections, naming decline is much less frequent, but is still reported in about 5% of patients [7, 8], highlighting the complexity and widespread distribution of the language network. Posterobasal temporal lobe regions are critically involved in naming function [5, 9] and auditory and picture naming fMRI robustly activated these language regions [10, 11]. Language fMRI can be used to lateralize the cortical activations associated with language function and predict the decline of naming function after ATLR [4, 6, 12] with high sensitivity and specificity, particularly when using naming fMRI paradigms [13]. Postoperative naming decline may occur even if language regions identified with direct cortical stimulation mapping are spared [14, 15], highlighting the role of subcortical white matter connections underpinning language function.

Diffusion MRI (dMRI)-based tractography can be used to visualize white matter fibre bundles, including language networks [16]. Changes of quantitative measures derived from diffusion tensor imaging (DTI), such as a decrease of fractional anisotropy (FA) or increase of mean diffusivity (MD), have been reported to reflect microstructural damage of language tracts and relate to impaired clinical language performance in temporal lobe epilepsy [17, 18]. Stronger preoperative lateralization of language tracts in the inferior frontal lobe to the dominant hemisphere was previously demonstrated to correlate with worse naming outcome after ATLR in a small cohort (*n* = 7) of adults with TLE [19]. Furthermore, a recent retrospective analysis showed that transection of the inferior fronto-occipital fasciculus (IFOF) during ATLR related to postsurgical naming decline [8].

We previously demonstrated that by combining methods of functional and structural connectivity, auditory and picture naming fMRI activation maxima can be used to delineate the structural connectivity of language regions in the temporal lobe in people with TLE [20]. In this work, we report the use of naming fMRI-guided tractography in people with TLE undergoing temporal lobe surgery. We hypothesized that the size and lateralization of preoperative language tracts in the dominant hemisphere would relate to postsurgical naming decline, with the ultimate goal to implement language tract visualization in presurgical planning as well as intraoperatively to improve language function outcome after epilepsy surgery.

## Methods

### Participants

We studied 20 patients with medically refractory unilateral TLE that represented a subgroup of our previously published cohort [20] who eventually proceeded to undergo epilepsy surgery at the National Hospital for Neurology and Neurosurgery (NHNN) between 2014 and 2018. All participants were fluent in written and spoken English. Exclusion criteria were contraindication to MRI, inability to give informed consent, or history of a focal to bilateral tonic–clonic seizure within 24 h prior to the scan. Demographic and clinical data are summarized in Table 1.

**Table 1:** Demographic data and descriptive statistics of clinical data of study population

Demographic data	N	%	
Sex (f/m)	9/11	45/55	
Handedness (l/r)	3/17	15/85	
TLE type (l/r)	9/11	45/55	
Surgery type (ATLR/lesionectomy)	16/4	80/20	
Education level (O-level/A-level/undergraduate/postgraduate)	7/6/5/2	35/30/25/10	

Descriptive statistics	N	Mean (SD)	Median	Range
Age (year)	20	37.95 (10.24)	37.50	19–58
Age at Onset of Epilepsy (year)	20	20.65 (13.48)	19.00	2–51
Epilepsy Duration (year)	20	16.80 (11.42)	13.50	0–42
Preoperative GNT	20	16.20 (4.79)	16.00	8–25
4-month postoperative GNT	20	14.65 (5.61)	16.00	1–23
12-month postoperative GNT	18	15.50 (5.45)	17.00	3–23

Demographic data are reported as n and percentage. Descriptive statistics are presented as mean (SD) and range

*ATLR* anterior temporal lobe resection, *f* female, *GNT* Graded Naming Test score, *l* left, *m* male, *r* right, *SD* standard deviation, *TLE* temporal lobe epilepsy

Prolonged EEG video telemetry confirmed and lateralized temporal lobe seizure onset in all patients. Structural MRI at 3 T identified hippocampal sclerosis (HS) in 11 patients (6 left/5 right), dual pathology (focal cortical dysplasia and HS) in 1 (right), dysembryoplastic neuroepithelial tumour (DNT) in 5 (2 left/3 right), low-grade glioma in 2 (1 left/1 right), and encephalocele in one patient (right). Lesions were located in the mesial temporal lobe in 18 patients (8 left/10 right) and in the lateral temporal lobe in two patients (1 left/1right). Handedness was assessed with the Edinburgh Hand Preference Inventory [21]. Sixteen patients (6 left/10 right) underwent standard en bloc ATLR, which included the resection of the hippocampus, and four patients (3 left/1 right) underwent temporal lobe lesionectomies.

### MR data acquisition

Imaging was performed on a 3 T General Electric Discovery MR750 scanner (GE, Wisconsin), using standard imaging gradients with a maximum strength of 50 mTm^−1^ and slew rate 200 Tm^−1^ s^−1^ and a 32-channel RF receive head array coil. For fMRI, gradient-echo planar T2*-weighted images (TE = 22 ms, TR = 2500 ms) with 50 contiguous 2.4 mm slices (0.1 mm gap) were acquired with a 24 × 24 cm field of view (FOV), 64 × 64 matrix, giving an in-plane pixel size of 3.75 × 3.75 mm. The FOV was positioned to maximize coverage of the frontal and temporal lobes and minimize signal drop-out from the temporal and orbitofrontal lobes and an Array Spatial Sensitivity Encoding Technique (ASSET) factor of 2 was used to mitigate geometric distortions.

Multi-shell dMRI data were acquired using a 2 mm isotropic single-shot spin echo sequence with a 256 × 256 mm FOV, 128 × 128 matrix and 70 slices (TR/TE = 7600/74.1 ms; ∂/Δ = 21.5/ 35.9 ms; parallel imaging acceleration factor 2.5) and a total of 115 volumes, with 11, 8, 32, and 64 gradient directions at b-values of 0, 300, 700, and 2500 s/mm^2^, respectively, and a single b = 0 reverse phase-encoding image. All participants additionally received a standard clinical TLE imaging protocol as described previously [20].

### Functional MRI tasks and processing

All participants performed two overt naming fMRI paradigms with active control conditions as described in detail previously [10, 11]: auditory naming (naming aloud objects from their auditory description; reversed speech as control condition, duration 5 min); and picture naming (naming aloud objects from their visual presentation; scrambled pictures and faces as control condition, duration 6.25 min).

fMRI data were analysed with Statistical Parametric Mapping 8 (http://www.fil.ion.ucl.ac.uk/spm/). Imaging time series of each subject were realigned, normalized into standard anatomical space using a scanner- and acquisition-specific template, and smoothed with a Gaussian kernel of 8 mm full-width at half-maximum.

At the first level, task-specific effects were estimated according to the general linear model [22]. Regressors of interest were formed by convolving blocks of stimuli with the canonical haemodynamic response function using motion parameters as regressors. Voxel-wise parameter estimates were calculated for the contrasts “auditory naming” (auditory naming vs. reversed speech) and “picture naming” (picture naming vs. scrambled pictures and faces).

At the second level, one-sample t-tests were used for main task effects across all participants. Activations are reported at *p* < 0.05, corrected for multiple comparisons (family-wise error rate [FWE] across the whole brain. Lateralization indices (LIs) were calculated using the formula LI = (L − R)/(L + R) with the bootstrap method of the lateralization index toolbox implemented in SPM8 [23] on the auditory naming and picture naming spmT maps based on anatomical temporal lobe masks created from the WFU PickAtlas in SPM8 [24] comprising superior, middle, inferior temporal gyrus and fusiform gyrus, and mesial temporal lobe structures as described in detail previously [10, 11]. An LI > 0.2 for left TLE and LI < -0.2 for right TLE were defined as having surgery on the language-dominant hemisphere for each fMRI task. For auditory naming, 9 patients were identified as language-dominant and 11 as non-dominant (1 bilateral, 10 contralateral) resections. For picture naming, 11 patients were identified as language-dominant and 9 as non-dominant (1 bilateral, 8 contralateral) resections.

### Diffusion MRI and tractography processing

dMRI data were corrected for scanner drift [25], motion, and susceptibility-induced distortion [26, 27] as described in detail previously [20]. A probabilistic tissue segmentation approach (Geodesic Information Flows; GIF) [28] was used to segment 3D-T1 images into white matter (WM), grey matter (GM), deep grey matter (dGM), and cerebrospinal fluid (CSF). Images were then transformed into an Anatomically Constrained Tractography (ACT) [29] five-tissue-type file and rigidly registered to dMRI space.

Seed regions for fibre tractography were defined in the left and right inferior temporal gyrus (auditory naming) and fusiform gyrus (picture naming) for each subject, created from individual peak fMRI activations during auditory and picture naming within a region of interest (ROI) defined from group activation maps of a previous study [10]. Individual peak fMRI activations and their homotopic correlates were registered into dMRI space, and using Euclidian distance maps, 100-voxel seed volumes in the WM region most adjacent to the cortical GM fMRI peak were created. The spatial variation of seed regions is displayed in online resource 1. Starting from the seed region, 1000 tracts were generated using whole-brain probabilistic tractography in MRTrix3 [30] with a minimum curvature radius of 1 mm, a step size of 1.0 mm, and a minimum fibre orientation distribution (FOD) amplitude of 0.1 and correction for total intracranial volume as described earlier [20]. Whole-brain tractography in a sample patient is displayed in online resource 2. Commonality maps were created from tract maps binarized at a 0.01 probability threshold that were averaged across groups, with a commonality value of 1 indicating an overlap of 100% of subjects having connections in the respective voxel and a value of 0 indicating that no subject has connections [16, 31, 32]. Tract volumes were extracted for the left and right hemisphere using these binary masks. LI of tract volumes were calculated to assess the extent of lateralization between the side of epilepsy surgery and the contralateral side using the formula [LI = (Vsurg − Vcontralat)/(Vsurg + Vcontralat)], where a positive LI indicates greater tract volume on the side of epilepsy surgery, and a negative index indicates greater tract volume on the contralateral side.

We performed a supplementary analysis using a single seed region based on group-level activation maxima coordinates of a previous study [7] to test whether this would yield similar results compared to individualized seeds. Group-level peak activation coordinates were transformed from a study-specific fMRI template to native diffusion space utilizing easyReg [33]. These were then dilated and used as seeds for tractography using the same methodology as in the main paper.

### Naming assessment and relation of tract measures to naming decline

As part of the patients’ routine presurgical neuropsychological assessments, naming performance was evaluated with the McKenna Graded Naming Test (GNT) [34], which was performed preoperatively, 4 months postoperatively, and 12 months postoperatively, and a decline of ≥ 4 items was considered clinically significant in line with previous work [4, 13, 35]. Two patients did not have 12-month naming scores due to loss of follow-up. For association with naming decline, left TLE and right TLE patients were combined by referencing the analyses to the laterality of surgical procedure rather than “left” and “right”.

Simple correlations of tract volumes and tract laterality indices with naming score change at 4-month and 12-month follow-up were investigated using Pearson correlation. Subsequently, parameters showing significant correlations with naming decline were entered into linear regression models, and postoperative naming score change was entered as a continuous variable. Linear regression models included preoperative naming scores, age at time of preoperative MRI, age at onset of epilepsy, epilepsy duration, birth sex, education status, and language fMRI LI as additional regressors to control for potential moderators of language outcome [36]. As patients may be stratified according to lateralization on fMRI, which impacts postoperative language outcome [8, 18], we performed post-hoc comparisons of correlations between patients identifying as language dominant (LI > 0.2) or non-dominant (LI < 0.2) on the respective fMRI task.

Additionally, a linear mixed effect model was used to assess the relationship of tract volumes and tract laterality indices with naming score change from preoperative to 4 months and 12 months postoperatively, with time point being included as a binary covariate in the model. Each individual subject was treated as a random effect. Models were compared to a “null” model, representing the regression without the tract volume or laterality, using chi-squared assessment. Marginal R^2^ of the model is reported. Supplementary analyses using z-transformed neuropsychology scores were performed.

## Results

### Demographic findings and language outcome

Demographic data and descriptive statistics are summarized in Table 1. At 4-month postoperative follow-up, 6 patients (4 left TLE, 2 right TLE) had a significant decline of naming scores (Table 2). Out of these 6 patients, 1 left TLE had no 12-month follow-up and 1 right TLE patient improved to a non-significant decline with the remaining 4 patients still presenting with a significant decline compared to preoperatively at 12-month follow-up (Table 2). All 4 patients with a significant decline at 12-month follow-up were identified as language-dominant to the side of surgery on preoperative picture naming fMRI. For auditory naming fMRI, three patients had identified as dominant and one patient as bilateral (online resource 3). No patient with a non-significant decline at 4-months developed a significant decline at 12-month follow-up.

**Table 2:** Contingency table of picture naming tract volume regression model for prediction of clinically significant naming decline (≥ 4 points on the McKenna Graded Naming Test) vs. actual outcome after temporal lobe surgery

	Actual decline	Actual no decline	Total
4-month follow-up			
Predicted decline	6	1	7
Predicted No decline	0	13	13
Total	6	14	20
12-month follow-up			
Predicted decline	4	1	5
Predicted no decline	0	13	13
Total	4	14	18

There were no significant differences between seizure-free or not seizure-free patients and significant naming decline at 4-month (Fisher’s exact test: 0.48) or 12-month follow-up (Fisher’s exact test: 0.49).

### fMRI results—main effects

**Fig. 1:**
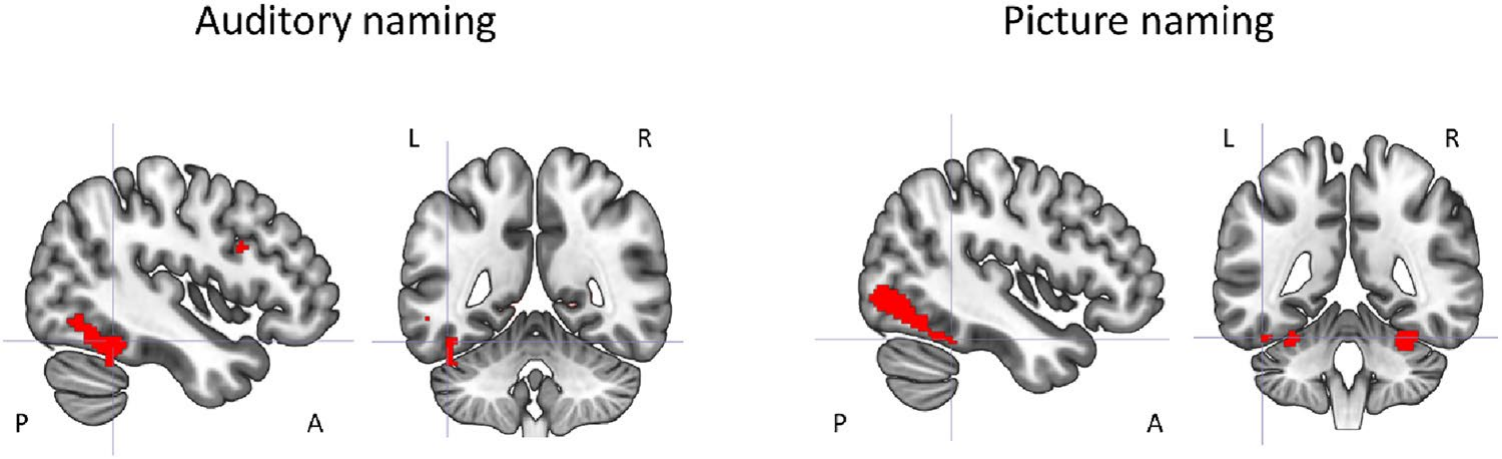
Main fMRI activation during auditory naming and picture naming across all participants shown superimposed on sagittal and coronal slices at *p* < 0.05, corrected for multiple comparisons (FWE). Crosshairs indicate activation maxima in the left inferior temporal gyrus (auditory naming) and left fusiform gyrus (picture naming)

During auditory naming, main activations across all subjects were seen in the left posterior inferior temporal gyrus, bilateral parahippocampal gyrus, left inferior frontal gyrus, left cuneus/precuneus and supramarginal gyrus, left middle and inferior occipital gyrus, and left cerebellum (Fig. 1, online resource 4). During picture naming, main activations were seen in the bilateral fusiform gyrus, bilateral middle occipital gyrus, and right cerebellum.

### Fibre tractography

**Fig. 2:**
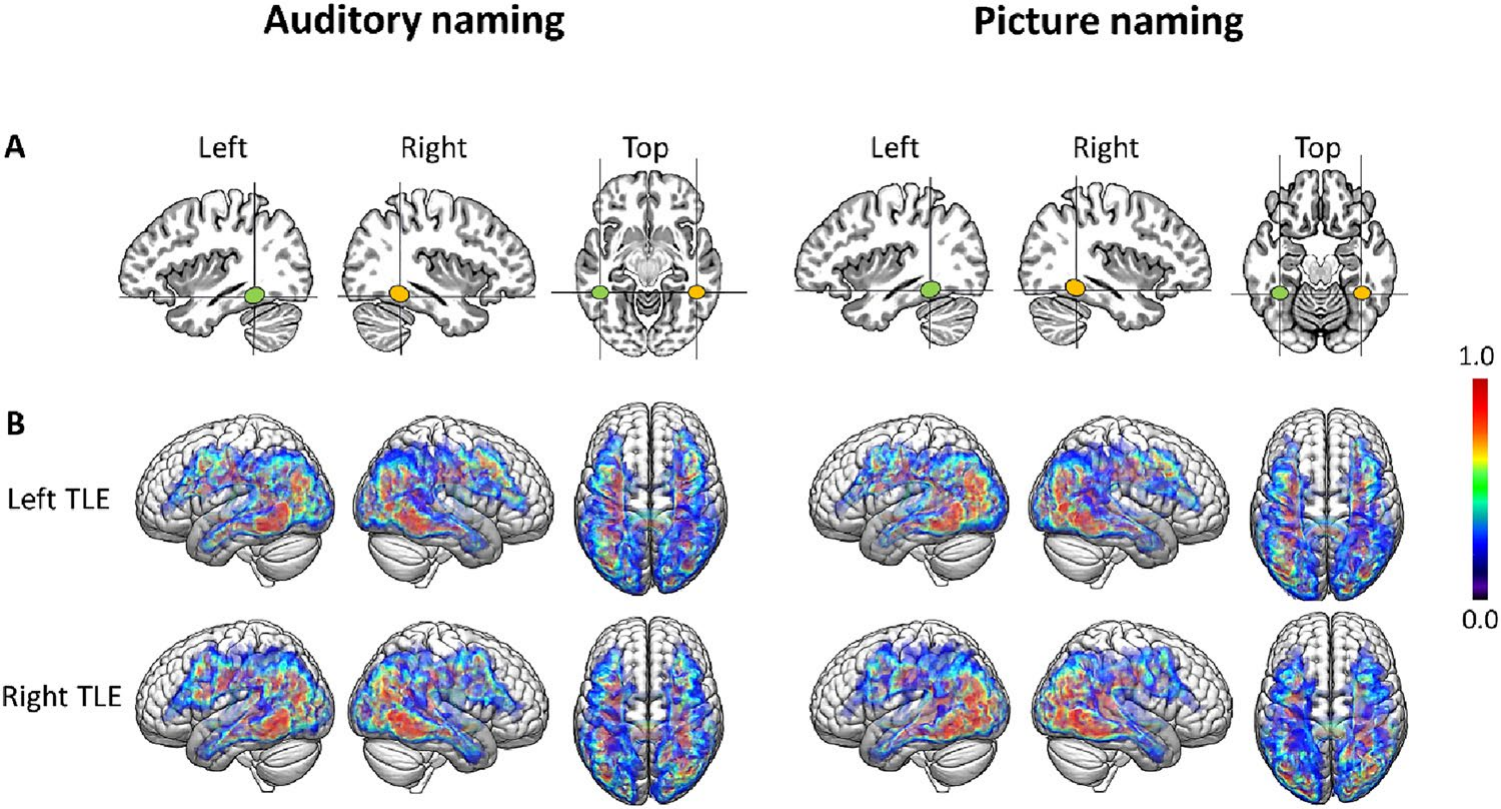
**A** Seed regions for tractography derived from fMRI activation maxima during auditory naming (left three columns) and picture naming (right three columns). **B** Group commonality maps (at a probability threshold of 0.01) of tracts seeding from the fMRI-guided seeds in the left posterobasal temporal lobe for left TLE and right TLE. Images show left, right, and top view surface renderings with embedded spatial distribution of tracts. The colour scale indicates the degree of overlap of tracts among subjects, expressed as a commonality value. A commonality value of 1 indicates an overlap of 100% of subjects having connections in the respective voxel, and a value of 0 indicates that no subject has connections. Similar connection patterns were observed for left TLE and right TLE, with consistent connections from the posterobasal temporal lobes to bilateral anterior temporal lobes, angular gyrus and occipital cortex, frontal and prefrontal regions

Group commonality maps of structural connections seeding from the bilateral posterior inferior temporal gyrus (auditory naming; Fig. 2) and left fusiform gyrus (picture naming; Fig. 2) showed that connections extended anteriorly to the temporal lobes including the temporal poles, and posteriorly to middle and inferior occipital gyri and precuneus, as well as to frontal and prefrontal regions. The distribution of tract volumes and laterality indices is displayed in online resource 5.

### Simple correlations with postoperative naming decline

**Fig. 3:**
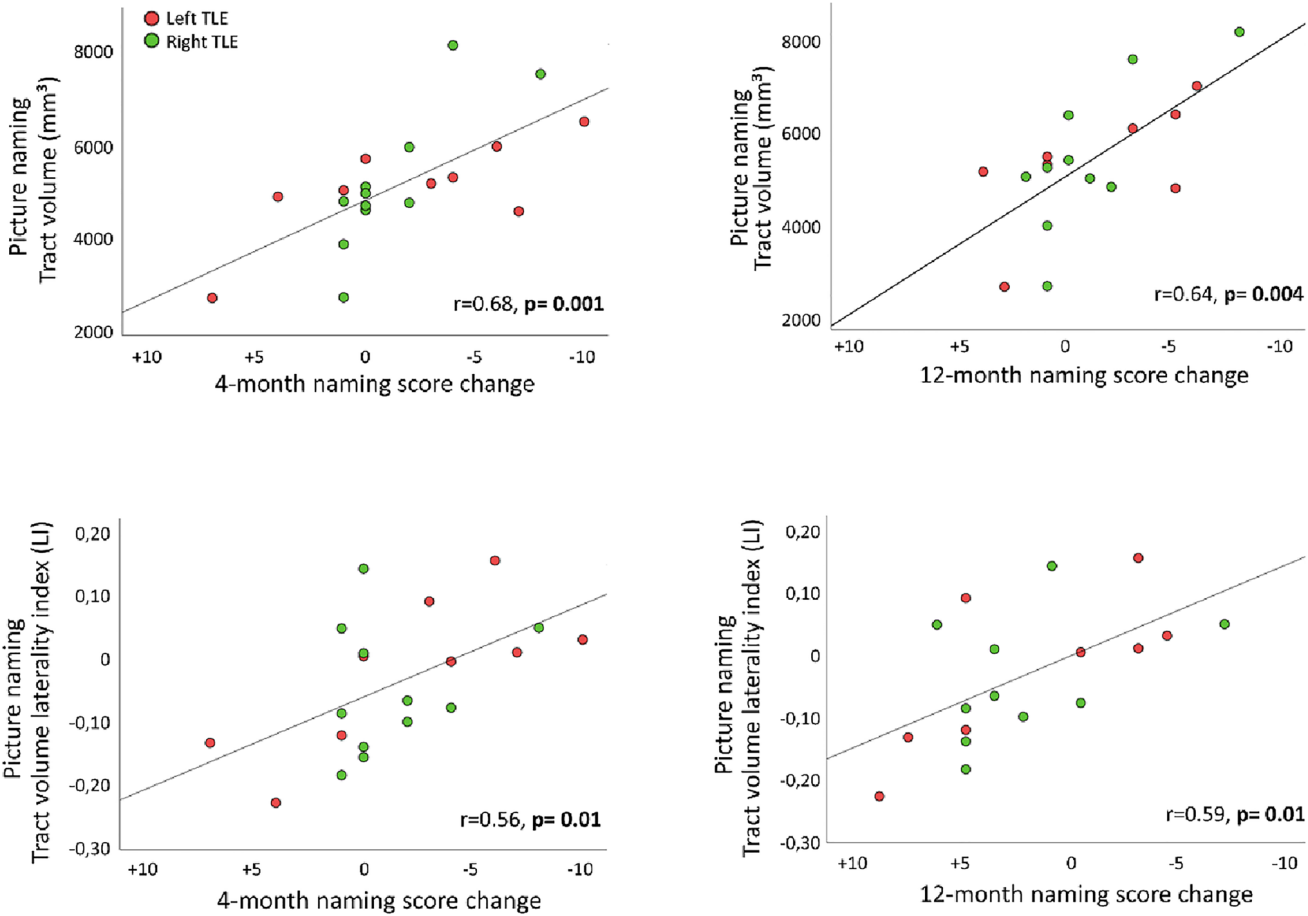
Correlation of picture naming tract volumes (upper row) and picture naming tract laterality indices (lower row) with naming decline at 4 months (left column) and 12 months (right column) after temporal lobe resection. Larger preoperative picture naming tract volumes in the hemisphere of subsequent temporal lobe resection correlated with greater postsurgical naming decline. Similarly, stronger preoperative lateralization of picture naming tracts to the side of surgery correlated with greater naming decline

For both auditory naming (r = 0.53, *p* = 0.02) and picture naming (r = 0.71, *p* < 0.001), larger preoperative tract volumes on the side of temporal lobe resection correlated with a more pronounced naming decline 4 months after surgery, which remained significant at 12-month follow-up for picture naming tract volumes only (r = 0.68, *p* = 0.002, Fig. 3) but not auditory naming (r = 0.42, *p* = 0.08).

Furthermore, stronger lateralization of tracts to the hemisphere of subsequent surgery also correlated with worse naming outcome for picture naming tracts at both 4-month (r = 0.56, *p* = 0.01) and 12-month follow-up (r = 0.59, *p* = 0.01, Fig. 3), and with a trend observed for auditory naming tracts at 4-month follow-up (r = 0.44, *p* = 0.052) and 12-month follow-up (r = 0.47, *p* = 0.052).

Comparing patients that identified as language fMRI dominant vs. fMRI non-dominant (bilateral or contralateral) on the respective fMRI task did not show any significant differences in correlations (all *p* > 0.05, see online resource 6a for full details). An additional analysis was performed where bilateral cases were treated as language-dominant, again showing no significant differences (all *p* > 0.05, online resource 6b).

### Multiple regression

The multiple regression model for picture naming tract volumes at 4-month follow-up was significant (F(9,10) = 3.77, *p* = 0.03, R^2^ = 0.77, adjusted R^2^ = 0.57), showing that a greater tract volume on the side of surgery was the strongest and only significant contributing factor (Beta = 1.10, *p* = 0.004) to postoperative naming outcome at 4-month follow-up, while all other clinical variables (age, age at onset of seizures, epilepsy duration, preoperative naming scores, surgery type, birth sex, education status, fMRI LI) remained non-significant (all *p* > 0.05, full details provided in online resource 7).

The regression model remained significant at 12-month follow-up (F(9,8) = 3.82, *p* = 0.04, R^2^ = 0.81, adjusted R^2^ = 0.60), again identifying greater ipsilateral tract volume as the only significant contributor to naming outcome (Beta = 0.94, *p* = 0.02), while all other clinical variables were non-significant (full details provided in online resource 7). Multiple regression models for auditory tract volume (4-month: F(9,10) = 1.00, *p* = 0.49; 12-month: F(9,8) = 1.65, *p* = 0.25) and for picture naming tract volume lateralization index (4-month: F(9,10) = 1.79, *p* = 0.11; 12-month: F(9,8) = 2.44, *p* = 0.11) were non-significant.

**Fig. 4:**
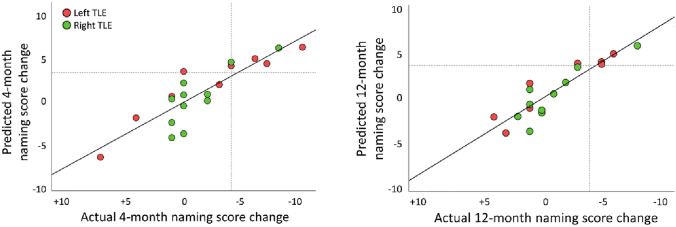
Correlation of predicted (multiple regression model) vs. actual change in naming score at 4 months (left panel) and 12 months after surgery (right panel). Dotted lines indicate the cutoff for clinically significant decline (≥ 4 points on the Graded Naming Test). The upper left quadrant shows patients who were predicted to develop a significant decline but who did not show a decline. All patients who developed a significant decline were correctly identified

Figure 4 demonstrates the relation of naming scores predicted by the regression models vs. the actual change in naming scores. Both the 4-month as well as 12-month regression models for picture naming tract volume gave a prediction of a significant decline of naming function with 100% sensitivity and 93% specificity (Table 2).

Supplementary analyses using linear mixed effect models were applied to assess whether the results could be modelled across both 4-month and 12-month decline. Greater tract volume to the ipsilateral hemisphere and tract lateralization were significantly related to the change in naming scores at 4- and 12-month follow-up both for auditory naming and picture naming. For picture naming tract volume and tract lateralization, results remained significant after accounting for preoperative naming scores, age, age at onset of seizures, epilepsy duration, surgery type, birth sex, education status, and language fMRI LI. Results of these analyses are summarized in online resource 8. Furthermore, linear mixed effect models were repeated based on group activation peak coordinates, which did not reveal significant effects for tract volumes and tract laterality indices (online resource 9).

## Discussion

Language decline after temporal lobe resection is a major concern for people with TLE, and the importance of white matter fibre bundles subserving language function for planning epilepsy surgery is increasingly acknowledged [8]. We combined functional and structural imaging methods, demonstrating that the preoperative volumes of white matter language tracts derived from picture naming fMRI maxima in the posterobasal temporal lobe relate to the extent of naming decline in people with TLE following temporal lobe resection 4 months postoperatively, with stable effects at 12-month follow-up.

Recent research highlights the functional relevance of white matter bundles in the basal temporal lobe, demonstrating that higher FA is linked to naming performance in left TLE, with indication for a functional reserve of the contralateral fusiform gyrus for postsurgical naming outcome [18]. Furthermore, a retrospective analysis in language fMRI-dominant temporal lobe resections demonstrated that damage to the IFOF was associated with poorer naming outcome [8]. The posterobasal temporal lobe is recognized as a “hub” for naming function [37], which is reliably activated during naming fMRI [10, 13]. Tracts seeding from these activation maxima reveal connection patterns that overlap with well-defined language fibre bundles, in particular inferior longitudinal fasciculus (ILF), IFOF, arcuate fasciculus (AF), middle longitudinal fasciculus (MLF), and uncinate fasciculus (UF) [20, 38, 39].

We confirm a previous report of language tract lateralization relating to postoperative naming decline [19] and extend on these findings by demonstrating that absolute tract volume, corrected for intracranial volume, ipsilateral to the side of subsequent temporal lobe resection seeding from the posterobasal temporal lobe determines a clinically significant naming decline with maximum sensitivity (100%) and high specificity (93%). This infers that more bilateral language tract connections, along with generally smaller volumes of language tracts ipsilateral to the hemisphere where surgery is performed, may be protective of postoperative language decline. Previous studies report that while word finding difficulties and naming decline are frequently observed in the first 6 months after temporal lobe surgery, language function may recover and even significantly improve after 1–5 years, particularly following non-dominant temporal lobe resection [40–43]. We investigated naming outcome both at 4 months as well as 12 months postoperatively, demonstrating robust effects at 12 months, irrespective of language fMRI lateralization status. Other potential moderators of language outcome like age, age at onset of seizures, epilepsy duration, birth sex, or education level [36] were also controlled for in our analyses.

Interestingly, absolute tract volume was identified as a more reliable and robust moderator of naming decline than tract lateralization, withstanding correction for potential modifiers of language outcome in a multivariate regression model. We argue that this may be attributable to the relatively bilateral distribution of language tracts. The low number of patients with pronounced lateralization of language tract volumes to the side where surgery was subsequently performed might not have allowed to explore this effect in a sufficient manner.

The finding that absolute tract volume more strongly relates to postoperative language deficits than tract laterality emphasizes that while longstanding epilepsy may lead to functional and structural reorganization to the contralateral hemisphere, patients with larger, dispersed interconnected language regions subserved by separate streamlines in the to-be-resected hemisphere are still at greater risk for language decline. This implies that the extent of resected tract volume may play a key role in the development of language deficits. A clinically relevant issue is whether modifying the surgical approach to avoid causing damage to relevant tracts, such as ILF and IFOF, may avert the development of naming difficulties after ATLR. This approach has been successfully implemented in the avoidance of visual field deficits after ATLR. It has been shown that changes in the surgical approach and intraoperative visualization of the optic radiation significantly reduces the risk of developing visual field deficits that prevent patients from driving [44].

Despite evidence for the implication of white matter fibre bundles in language function in TLE, translation into individual surgical approaches in epilepsy surgery remains limited. Along with recent findings in a retrospective cohort [8], our results suggest the possibility that through the identification of critical tracts for language, individualized surgical approaches avoiding these tracts may prevent clinically relevant postsurgical naming deficits, which is now the subject of an ongoing prospective study. Of note, our findings were not reproducible when using a group average tractography seed based on previously published naming fMRI data as compared to individualized fMRI-based seeds, highlighting the benefits of personalized diagnostic approaches in presurgical epilepsy evaluation. However, future studies with larger cohorts should continue to explore the possibility to use predefined fMRI coordinates as seeding points, as this might open the possibility to remove language fMRI from the presurgical protocol in selected cases, e.g. patients who require sedation for MRI due to claustrophobia, or where language barrier or pre-existing cognitive impairments limit the informative value of fMRI.

The association of language tract volumes with postoperative naming decline in this study was compatible to our previous findings with naming fMRI [13], also confirming better results for picture naming-based results compared to auditory naming. Language fMRI generally shows high concordance with direct cortical stimulation mapping [45, 46], and it has been suggested that the extent of resection of fMRI activation during a word definition decision task impacts postoperative naming ability outcome [43]. However, language deficits may still occur even when brain regions with activations during presurgical language fMRI are spared [47, 48].

The main limitation of this study is its small sample, which limits generalizability. On the other hand, using language fMRI paradigms to guide tractography analysis was likely responsible to achieve high specificity with 100% sensitivity for detecting language decline. The heterogeneity of our sample, with about half of our patients having HS, and including other causes of TLE like FCD, was both a limitation, as we could not perform subgroup analyses due to insufficient sample sizes, but also a strength as it increases generalizability across different pathologies. While mechanisms underlying TLE differ in patients with stable lesions like HS from slowly evolving lesions like low-grade glioma, this work focuses on the affection of language-relevant white matter tracts, regardless of the cause of epilepsy.Future studies in larger samples are warranted to confirm and extend on our fMRI-guided tractography findings for translation into clinical practice, with particular respect to various underlying pathologies of TLE.

### Clinical impact and relevance

Our findings shed new light on the functional relevance of white matter tracts underpinning language in TLE, and the importance to visualize structural connections to mitigate functional damage after epilepsy surgery. Follow-up studies in well-powered samples are warranted to investigate the relationship between the extent of resected fMRI-based language tracts and naming outcome after surgery, with the inference that individualized surgical approaches, lesion mapping, and intraoperative visualization of language tracts may prevent postoperative decline as well as influence postoperative recovery or improvement of naming function in people with TLE.

### Supplementary Information

Below is the link to the electronic supplementary material.

Supplementary file1 (DOCX 1367 KB)

## Declarations

### Data availability

Anonymized data can be made available to qualified investigators upon reasonable request to the corresponding author.
