# Supplementary material for: Naming fMRI-guided white matter language tract volumes influence naming decline after temporal lobe resection
Source: J Neurol. 2024 Apr 7;271(7):4158–67. doi: 10.1007/s00415-024-12315-2 (PMC11233363; doi:10.1007/s00415-024-12315-2)
Supplement: Supplementary file 1 — Supplementary file1 (DOCX 1367 KB) [file 415_2024_12315_MOESM1_ESM.docx]

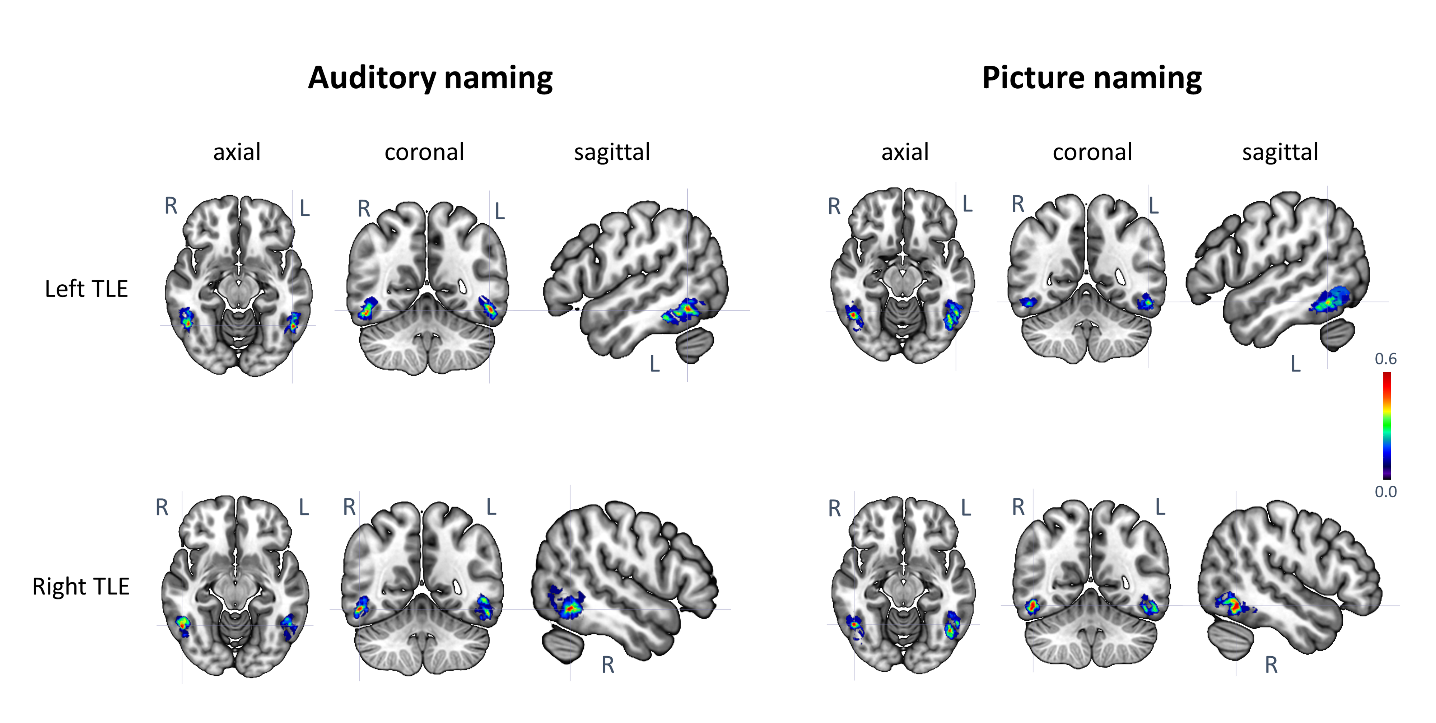


Online resource 1. Group commonality maps of seed regions for tractography in the left and right posterobasal temporal lobe for left TLE and right TLE. Images show axial, coronal and sagittal slices with embedded spatial distribution of seed regions. The colour scale indicates the degree of overlap of seed regions among subjects, expressed as a commonality value. A commonality value of 1 indicates an overlap of 100% of subjects having part of the seed region in the respective voxel.


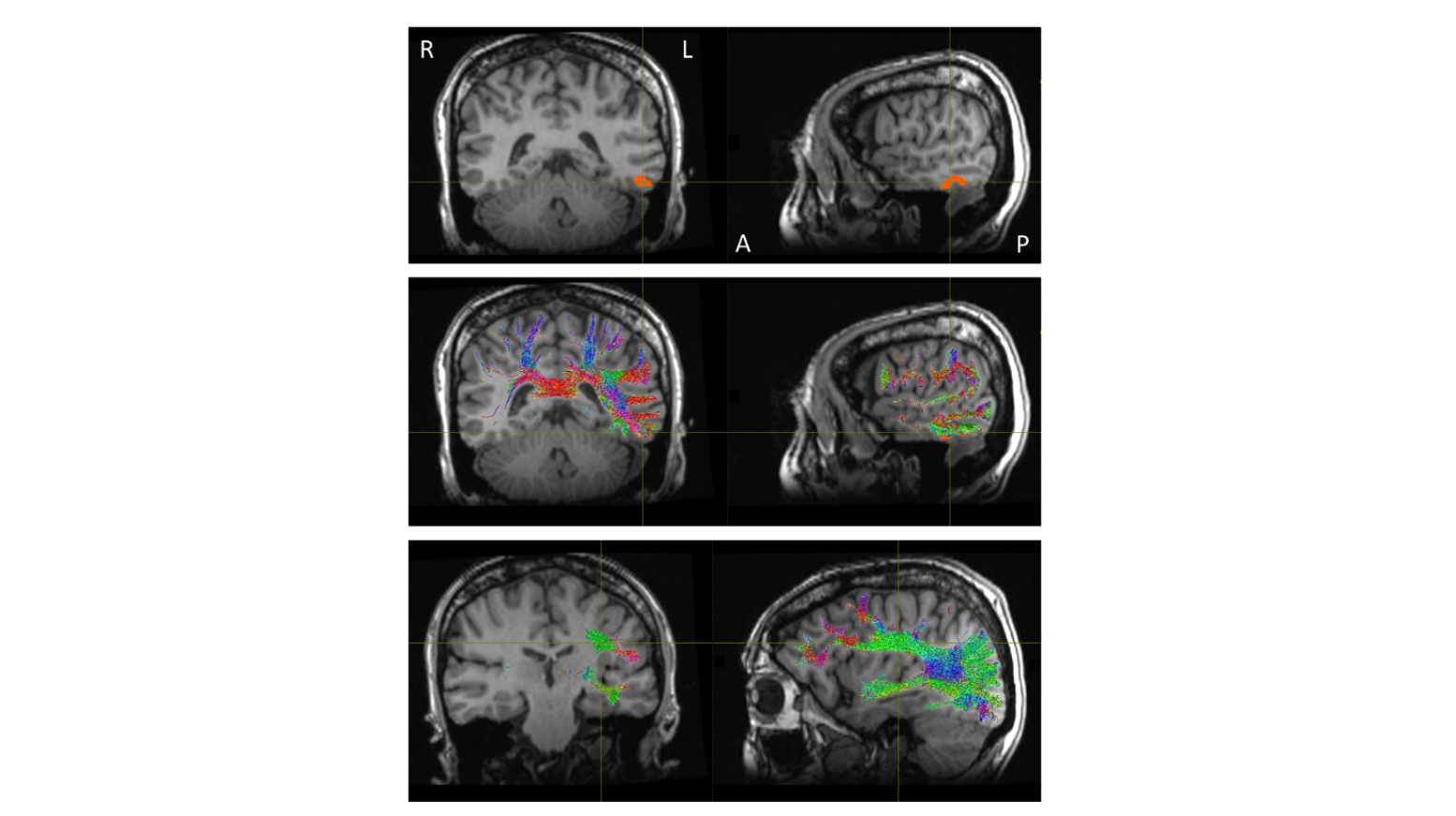


Online resource 2. Whole brain tractography in a sample patient. Upper row: White matter seed region adjacent to picture naming fMRI maximum used as starting point for whole-brain tractography. Middle row: Fibre tracts with direction colour-coding (green: anterior-to-posterior, blue: craniocaudal, red: right-to-left) in the plane of the seed region. Lower row: Fibre tracts in different plane view. Note: A = anterior; L = left, P = posterior, R = right

Online resource 3. Individual Demographic data and descriptive statistics of clinical data of study population.

| Subject | Age | Sex | Epilepsy duration | Age at Onset | AN fMRI | PN fMRI | TLE side | TLE location | Surgical procedure | 4-month  seizure outcome | 12-month seizure outcome | 4-month naming score change | 12-month naming score change |
| --- | --- | --- | --- | --- | --- | --- | --- | --- | --- | --- | --- | --- | --- |
| 1 | 51 | female | 33 | 18 | non-dominant | dominant | left | mesial | ATLR | ILAE 1 | ILAE 1 | -7,00 | -5,00 |
| 2 | 19 | female | 0 | 19 | dominant | dominant | left | mesial | ATLR | ILAE 1 | ILAE 1 | -6,00 | -5,00 |
| 3 | 32 | male | 13 | 19 | dominant | dominant | left | lateral | lesionectomy | ILAE 1 | ILAE 1 | -4,00 | n.a. |
| 4 | 32 | male | 25 | 7 | non-dominant | non-dominant | left | mesial | ATLR | ILAE 1 | ILAE 1 | +7,00 | +3,00 |
| 5 | 30 | male | 6 | 24 | non-dominant | non-dominant | left | mesial | ATLR | ILAE 1 | ILAE 1 | +1,00 | +1,00 |
| 6 | 38 | female | 36 | 2 | dominant | dominant | left | mesial | ATLR | ILAE 4 | ILAE 4 | -3,00 | +1,00 |
| 7 | 23 | male | 9 | 14 | dominant | dominant | left | mesial | lesionectomy | ILAE 1 | ILAE 1 | -10,00 | -6,00 |
| 8 | 34 | male | 13 | 21 | dominant | dominant | left | mesial | lesionectomy | ILAE 1 | ILAE 2 | 0,00 | -3,00 |
| 9 | 48 | male | 42 | 6 | non-dominant | non-dominant | left | mesial | ATLR | ILAE 1 | ILAE 1 | +4,00 | +4,00 |
| 10 | 46 | female | 7 | 39 | non-dominant | dominant | right | mesial | ATLR | ILAE 1 | ILAE 3 | -4,00 | -3,00 |
| 11 | 38 | male | 8 | 30 | dominant | dominant | right | lateral | lesionectomy | ILAE 1 | ILAE 4 | -2,00 | -1,00 |
| 12 | 45 | male | 31 | 14 | non-dominant | non-dominant | right | mesial | ATLR | ILAE 1 | ILAE 1 | +1,00 | +1,00 |
| 13 | 49 | male | 14 | 35 | non-dominant | non-dominant | right | mesial | ATLR | ILAE 1 | ILAE 1 | 0,00 | 0,00 |
| 14 | 24 | female | 17 | 7 | dominant | dominant | right | mesial | ATLR | ILAE 1 | ILAE 3 | -8,00 | -8,00 |
| 15 | 45 | male | 4 | 41 | non-dominant | non-dominant | right | mesial | ATLR | ILAE 1 | ILAE 1 | -2,00 | 0,00 |
| 16 | 37 | female | 25 | 2 | non-dominant | non-dominant | right | mesial | ATLR | ILAE 1 | ILAE 1 | 0,00 | -2,00 |
| 17 | 44 | female | 21 | 23 | dominant | dominant | right | mesial | ATLR | ILAE 1 | ILAE 1 | 0,00 | n.a. |
| 18 | 30 | female | 16 | 14 | dominant | dominant | right | mesial | ATLR | ILAE 1 | ILAE 2 | 0,00 | +1,00 |
| 19 | 36 | female | 9 | 27 | non-dominant | non-dominant | right | mesial | ATLR | ILAE 1 | ILAE 1 | +1,00 | +1,00 |
| 20 | 58 | male | 7 | 51 | non-dominant | non-dominant | right | mesial | ATLR | ILAE 3 | ILAE 3 | +1,00 | +2,00 |

Online resource 4. Coordinates and Z-scores of whole-brain fMRI activation during auditory and picture naming during auditory naming, picture naming and verbal fluency shown corrected for multiple comparisons (FWE; p < 0.05).

| **Whole brain fMRI activations** | | | | | | | | |
| --- | --- | --- | --- | --- | --- | --- | --- | --- |
|  | **Auditory naming** | | | | **Picture naming** | | | |
|  | **Left** | | **Right** | | **Left** | | **Right** | |
|  | Z | Coordinates | Z | Coordinates | Z | Coordinates | Z | Coordinates |
| Inferior frontal gyrus | 4.18 | -50 16 24 |  |  |  |  |  |  |
|  | 4.18 | -50 32 2 |  |  |  |  |  |  |
| Inferior temporal gyrus | 4.47 | -44 -44 -20 |  |  |  |  |  |  |
| Fusiform gyrus |  |  |  |  | 4.45 | -42 -44 -24 | 5.20 | 42 -54 12 |
| Parahippocampal gyrus | 4.88 | -20 -50 2 | 4.16 | 12 -44 -4 |  |  |  |  |
| Cuneus/Calcarine | 4.75 | -8 -90 4 |  |  |  |  |  |  |
| Precuneus | 4.38 | -4 -52 10 |  |  |  |  |  |  |
| Supramarginal gyrus | 4.34 | -54 26 18 |  |  |  |  |  |  |
| Cerebellum | 3.96 | -10 -52 -10 |  |  |  |  | 5.31 | 26 -46 -26 |
| Middle occipital gyrus | 4.12 | -26 -76 4 |  |  | 5.04 | -40 -74 0 | 5.22 | 28 -94 2 |
| Inferior occipital gyrus | 4.08 | -42 -66 -6 |  |  |  |  |  |  |


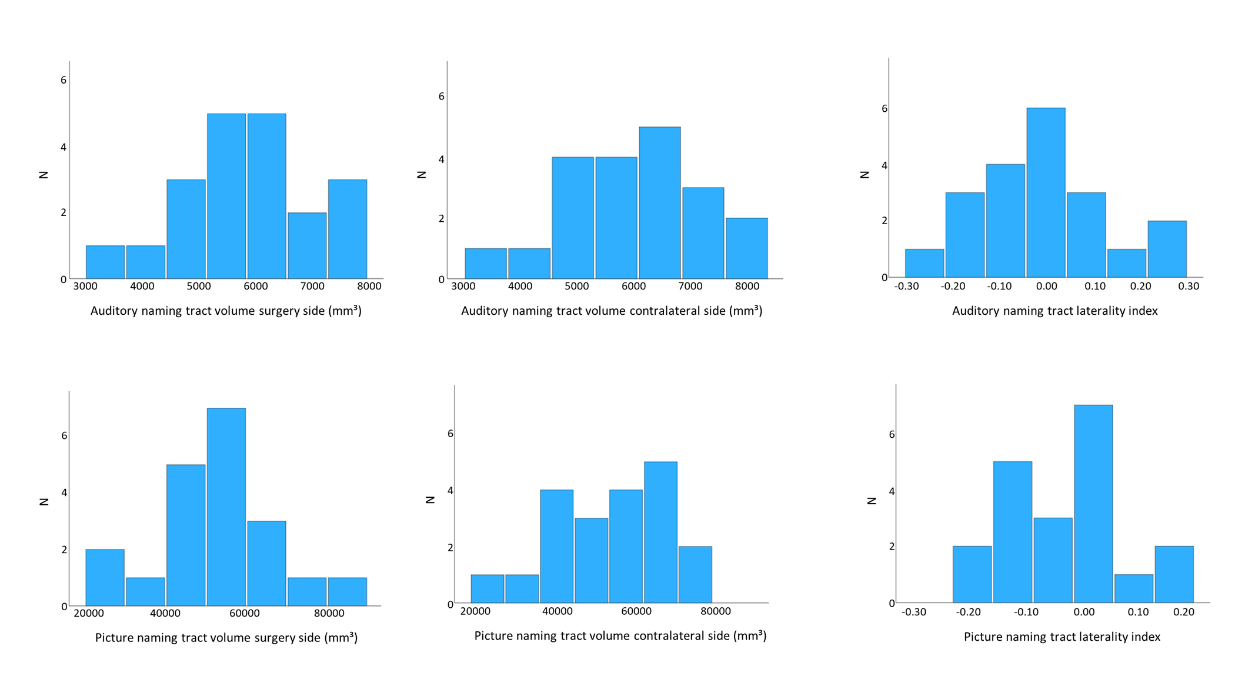


Online resource 5. Distribution of auditory naming (upper row) and picture naming (lower row) tract volumes and tract laterality indices.

Online resource 6a. Comparison of correlation coefficients (Fisher r-to-z transformation) between patients classified as fMRI dominant vs. fMRI non-dominant (bilateral or contralateral) resections.

|  | ***r* value**  **fMRI dominant (n)** | ***r* value**  **fMRI non-dominant (n)** | **z** | **p** |
| --- | --- | --- | --- | --- |
| **4-month follow up** |  |  |  |  |
| Auditory naming Tract volume | 0.44 (9) | 0.31 (11) | 0.28 | 0.77 |
| Picture naming Tract volume | 0.48 (11) | 0.82 (9) | -1.17 | 0.24 |
| Picture naming  Tract LI | 0.43 (11) | 0.58 (9) | -0.38 | 0.70 |
| **12-month follow-up** |  |  |  |  |
| Picture naming  Tract volume | 0.52 (9) | 0.72 (9) | -0.57 | 0.57 |
| Picture naming  Tract LI | 0.27 (9) | 0.73 (9) | -1.13 | 0.26 |
|  |  |  |  |  |
|  |  |  |  |  |

Online resource 6b. Comparison of correlation coefficients (Fisher r-to-z transformation) between patients classified as fMRI dominant (ipsilateral or bilateral) vs. fMRI non-dominant (contralateral) resections.

|  | ***r* value**  **fMRI dominant (n)** | ***r* value**  **fMRI non-dominant (n)** | **z** | **p** |
| --- | --- | --- | --- | --- |
| **4-month follow up** |  |  |  |  |
| Auditory naming Tract volume | 0.56 (10) | 0.48 (10) | -0.06 | 0.95 |
| Picture naming Tract volume | 0.52 (12) | 0.82 (8) | -1.04 | 0.30 |
| Picture naming  Tract LI | 0.26 (12) | 0.74 (8) | -1.23 | 0.22 |
| **12-month follow-up** |  |  |  |  |
| Picture naming  Tract volume | 0.49 (10) | 0.74 (8) | -0.71 | 0.48 |
| Picture naming  Tract LI | 0.23 (10) | 0.81 (8) | -1.52 | 0.13 |

Online resource 7. Linear regression results for picture naming tract volumes

|  | **4-month follow up** | | |  | **12-month follow up** | | |
| --- | --- | --- | --- | --- | --- | --- | --- |
|  | Beta | 95% CI | p |  | Beta | 95% CI | p |
| Picture naming Tract volume | 1.10 | 0.45–1.76 | **0.004** |  | 0.94 | 0.23–1.57 | **0.02** |
| Age | -1.51 | -3.72–0.70 | 0.16 |  | 0.41 | -1.83–2.62 | 0.69 |
| Age at onset | 1.80 | -0.87–4.48 | 0.16 |  | -0.59 | -3.23–2.13 | 0.65 |
| Epilepsy duration | 1.53 | -0.92–3.97 | 0.19 |  | -0.75 | -3.16–1.74 | 0.52 |
| Sex | -0.26 | -1.09–0.58 | 0.50 |  | 0.22 | -0.67–1.11 | 0.58 |
| Surgery type | .0.02 | -0.67–0.62 | 0.94 |  | 0.40 | -0.25–1.10 | 0.18 |
| Education status | 0.36 | -0.24–0.95 | 0.21 |  | 0.47 | -0.13–1.06 | 0.11 |
| Preoperative naming score | -0.347 | -0. 99–0.05 | 0.07 |  | -0.46 | -0.98–0.08 | 0.09 |
| Language fMRI LI | -0.57 | -1.23–0.15 | 0.11 |  | 0.01 | -0.73–0.75 | 0.98 |

Online resource 8a. Linear mixed effect model results for tract volumes and tract laterality indices with naming score change from preoperative to 4-month and 12-month postoperatively.

| **Auditory naming Tract volume & change in raw naming scores** |  | **Statistics** | |
| --- | --- | --- | --- |
| Auditory naming Tract volume |  | 𝛽=-1.767, p=0.026, 95%CI=-3.190:-0.342 | |
| Time |  | 𝛽=-0.477, p=0.330, 95% CI=-0.471:1.442 | |
| Patient random effects |  | Variance=9.020, SD=3.003 | |
| Overall Model Performance |  | X^2^(1) 5.639, p = 0.018, Marginal R^2^ = 0.22 | |
|  |  |  | |
| **Picture naming Tract volume & change in raw naming scores** |  | **Statistics** | |
| Picture naming Tract volume |  | 𝛽=-2.538, p < 0.001, 95% CI=-3.683:-1.395 | |
| Time |  | 𝛽=-0.464, p=0.341, 95% CI=-0.482:1.419 | |
| Patient random effects |  | Variance=5.494, SD=2.344 | |
| Overall Model Performance |  | X^2^(1)=14.285, p < 0.001, Marginal R^2^=0.47 | |
|  |  |  | |
| **Auditory naming Tract LI & change in raw naming scores** |  | **Statistics** | |
| Auditory naming Tract LI |  | 𝛽=-1.617, p=0.045, 95% CI=-3.079:-0.151 |  |
| Time |  | 𝛽=-0.483, p=0.325, 95% CI=-0.465:1.451 | |
| Patient random effects |  | Variance=9.550, SD=3.090 | |
| Overall Model Performance |  | X^2^(1)=4.583, p=0.032, Marginal R^2^=0.18 |  |
|  |  |  | |
| **Picture naming Tract LI & change in raw naming scores** |  | **Statistics** | |
| Picture naming Tract LI |  | 𝛽=-2.092, p < 0.001, 95% CI=-3.420:-0.762 | |
| Time |  | 𝛽=-0.473, p=0.333, 95% CI=-0.473:1.434 | |
| Patient random effects |  | Variance=7.727, SD=2.780 | |
| Overall Model Performance |  | X^2^(1)=8.432, p=0.004, Marginal R^2^=0.31 | |
|  |  |  | |
|  |  |  | |
| **Auditory naming Tract volume & change in z- naming scores** |  | **Statistics** | |
| Auditory naming Tract volume |  | 𝛽=-0.490, p=0.020, 95% CI=-0.864:-0.114 | |
| Time |  | 𝛽=0.123, p=0.388, 95% CI=-0.153:0.406 | |
| Patient random effects |  | Variance=0.608, SD=0.780 | |
| Overall Model Performance |  | X^2^(1)=6.153, p=0.013, Marginal R^2^=0.23 | |
|  |  |  | |
| **Picture naming Tract volume & change in z- naming scores** |  | **Statistics** | |
| Picture naming Tract volume |  | 𝛽=-0.693, p < 0.001, 95% CI=-0.990:-0.396 | |
| Time |  | 𝛽=0.120, p=0.397, 95% CI=-0.155:0.401 | |
| Patient random effects |  | Variance=0.352, SD=0.593 | |
| Overall Model Performance |  | X^2^(1)=15.311, p < 0.001, Marginal R^2^=0.48 | |
|  |  |  | |
| **Auditory naming Tract LI & change in z- naming scores** |  | **Statistics** | |
| Auditory naming Tract LI |  | 𝛽=-0.421, p=0.051, 95% CI=-0.814:-0.027 | |
| Time |  | 𝛽=0.124, p=0.386, 95% CI=-0.153:0.408 | |
| Patient random effects |  | Variance=0.674, SD=0.821 | |
| Overall Model Performance |  | X^2^(1)=4.333, p=0.037, Marginal R^2^=0.17 | |
|  |  |  | |
| **Picture naming Tract LI & change in z- naming scores** |  | **Statistics** | |
| Picture naming Tract LI |  | 𝛽=-0.571, p=0.005, 95% CI=-0.922:-0.220 | |
| Time |  | 𝛽=0.122, p=0.391, 95% CI=-0.154:0.404 | |
| Patient random effects |  | Variance=0.520, SD=0.721 | |
| Overall Model Performance |  | X^2^(1)=8.917, p=0.003, Marginal R^2^=0.32 | |

Online resource 8b. Linear mixed effect model results for tract volumes and tract laterality indices with naming score change from preoperative to 4-month and 12-month postoperatively, accounting for preoperative language fMRI LI, preoperative naming scores, age, age at onset of seizures, epilepsy duration, surgery type, birth sex, and education status.

| **Auditory naming Tract volume & change in raw naming scores** |  | **Statistics** |
| --- | --- | --- |
| Auditory naming Tract volume |  | 𝛽 = -1.214, p = 0.35, 95CI= -3.022:0.599 |
| Auditory Naming fMRI LI |  | 𝛽 = 0.35, p = 0.707, 95CI= -0.966:1.669 |
| Preoperative Scores |  | 𝛽 = 0.028, p = 0.897, 95CI= -0.276:0.33 |
| Gender |  | 𝛽 = -3.259, p = 0.163, 95CI= -6.434:-0.102 |
| Operation Type |  | 𝛽 = -3.902, p = 0.248, 95CI= -8.545:0.747 |
| Epilepsy Duration |  | 𝛽 = 0.272, p = 0.96, 95CI= -7.519:8.052 |
| Age of Onset |  | 𝛽 = 0.148, p = 0.98, 95CI= -8.389:8.668 |
| Education |  | 𝛽 = -0.199, p = 0.868, 95CI= -1.903:1.5 |
| Age |  | 𝛽 = 0.047, p = 0.917, 95CI= -0.603:0.698 |
| Time |  | 𝛽 = 0.447, p = 0.362, 95CI= -0.503:1.401 |
| Patient random effects |  | Variance=10.27, SD=3.205 |
| Overall Model Performance |  | X^2^(1)=1.807, p=0.179, Marginal R^2^=0.36 |

| **Picture naming Tract volume & change in raw naming scores** |  | **Statistics** |
| --- | --- | --- |
| Picture naming Tract volume |  | 𝛽 = -3.794, p = 0.004, 95CI= -5.253:-2.324 |
| Picture Naming fMRI LI |  | 𝛽 = 1.249, p = 0.288, 95CI= -0.378:2.853 |
| Preoperative Scores |  | 𝛽 = 0.358, p = 0.057, 95CI= 0.117:0.6 |
| Gender |  | 𝛽 = 0.663, p = 0.799, 95CI= -3.076:4.313 |
| Operation Type |  | 𝛽 = -1.372, p = 0.584, 95CI= -4.94:2.116 |
| Epilepsy Duration |  | 𝛽 = -2.024, p = 0.602, 95CI= -7.466:3.428 |
| Age of Onset |  | 𝛽 = -2.826, p = 0.508, 95CI= -8.786:3.136 |
| Education |  | 𝛽 = -1.486, p = 0.135, 95CI= -2.812:-0.165 |
| Age |  | 𝛽 = 0.25, p = 0.469, 95CI= -0.232:0.73 |
| Time |  | 𝛽 = 0.406, p = 0.403, 95CI= -0.574:1.313 |
| Patient random effects |  | Variance=4.028, SD=2.007 |
| Overall Model Performance |  | X^2^(1)=17.656, p < 0.001, Marginal R^2^=0.62 |

| **Auditory naming Lateralisation Index & change in raw naming scores** |  | **Statistics** |
| --- | --- | --- |
| Auditory naming LI |  | 𝛽= -1.878, p = 0.065, 95CI= -3.241:-0.561 |
| Auditory Naming fMRI LI |  | 𝛽= 1.252, p = 0.19, 95CI= -0.034:2.57 |
| Preoperative Scores |  | 𝛽= -0.195, p = 0.277, 95CI= -0.446:0.05 |
| Gender |  | 𝛽= -3.209, p = 0.096, 95CI= -5.744:-0.631 |
| Operation Type |  | 𝛽= -6.757, p = 0.033, 95CI= -10.713:-2.781 |
| Epilepsy Duration |  | 𝛽= -0.435, p = 0.924, 95CI= -6.831:6 |
| Age of Onset |  | 𝛽= -0.557, p = 0.909, 95CI= -7.421:6.366 |
| Education |  | 𝛽= -0.527, p = 0.622, 95CI= -2.038:0.972 |
| Age |  | 𝛽= 0.036, p = 0.926, 95CI= -0.511:0.578 |
| Time |  | 𝛽= 0.484, p = 0.325, 95CI= -0.428:1.489 |
| Patient random effects |  | Variance=7.614, SD=2.759 |
| Overall Model Performance |  | X^2^(1)=7.085, p=0.008, Marginal R^2^=0.46 |

| **Picture naming Lateralisation Index & change in raw naming scores** |  | **Statistics** |
| --- | --- | --- |
| Picture naming LI |  | 𝛽= -2.067, p = 0.043, 95CI= -3.372:-0.755 |
| Picture Naming fMRI LI |  | 𝛽= -0.745, p = 0.555, 95CI= -2.527:1.03 |
| Preoperative Scores |  | 𝛽= -0.098, p = 0.538, 95CI= -0.323:0.126 |
| Gender |  | 𝛽= -3.776, p = 0.17, 95CI= -7.528:-0.054 |
| Operation Type |  | 𝛽= -5.637, p = 0.065, 95CI= -9.611:-1.673 |
| Epilepsy Duration |  | 𝛽= -2.72, p = 0.589, 95CI= -9.801:4.382 |
| Age of Onset |  | 𝛽= -3.487, p = 0.527, 95CI= -11.229:4.274 |
| Education |  | 𝛽= -0.736, p = 0.511, 95CI= -2.311:0.838 |
| Age |  | 𝛽= 0.239, p = 0.584, 95CI= -0.377:0.852 |
| Time |  | 𝛽= 0.439, p = 0.369, 95CI= -0.512:1.377 |
| Patient random effects |  | Variance=6.987, SD=2.643 |
| Overall Model Performance |  | X^2^(1)=8.491, p=0.004, Marginal R^2^=0.49 |

| **Auditory naming Tract volume & change in naming Z-scores** |  | **Statistics** |
| --- | --- | --- |
| Auditory naming Tract volume |  | 𝛽= -0.182, p = 0.586, 95CI= -0.653:0.291 |
| Auditory Naming fMRI LI |  | 𝛽= 0.014, p = 0.952, 95CI= -0.325:0.355 |
| Preoperative Z-scores |  | 𝛽= -0.115, p = 0.561, 95CI= -0.394:0.162 |
| Gender |  | 𝛽= -0.98, p = 0.103, 95CI= -1.783:-0.184 |
| Operation Type |  | 𝛽= -1.277, p = 0.145, 95CI= -2.453:-0.097 |
| Epilepsy Duration |  | 𝛽= 0.316, p = 0.822, 95CI= -1.671:2.3 |
| Age of Onset |  | 𝛽= 0.26, p = 0.866, 95CI= -1.921:2.437 |
| Education |  | 𝛽= -0.032, p = 0.916, 95CI= -0.468:0.4 |
| Age |  | 𝛽= -0.018, p = 0.882, 95CI= -0.185:0.15 |
| Time |  | 𝛽= 0.111, p = 0.434, 95CI= -0.164:0.392 |
| Patient random effects |  | Variance=0.648, SD=0.805 |
| Overall Model Performance |  | X^2^(1)=0.612, p=0.434, Marginal R^2^=0.39 |
| **Picture naming Tract volume & change in naming Z-scores** |  | **Statistics** |
| Picture naming Tract volume |  | 𝛽= -0.972, p = 0.014, 95CI= -1.442:-0.499 |
| Picture Naming fMRI LI |  | 𝛽= 0.359, p = 0.291, 95CI= -0.112:0.823 |
| Preoperative Scores |  | 𝛽= 0.264, p = 0.186, 95CI= -0.006:0.536 |
| Gender |  | 𝛽= 0.059, p = 0.937, 95CI= -1.016:1.107 |
| Operation Type |  | 𝛽= -0.502, p = 0.494, 95CI= -1.539:0.517 |
| Epilepsy Duration |  | 𝛽= -0.476, p = 0.667, 95CI= -2.033:1.083 |
| Age of Onset |  | 𝛽= -0.731, p = 0.552, 95CI= -2.453:0.991 |
| Education |  | 𝛽= -0.362, p = 0.19, 95CI= -0.737:0.01 |
| Age |  | 𝛽= 0.063, p = 0.531, 95CI= -0.078:0.203 |
| Time |  | 𝛽= 0.1, p = 0.48, 95CI= -0.184:0.367 |
| Patient random effects |  | Variance=0.303, SD=0.550 |
| Overall Model Performance |  | X^2^(1)=12.832, p < 0.001, Marginal R^2^=0.60 |

| **Auditory naming Lateralisation Index & change in naming Z-scores** |  | **Statistics** |
| --- | --- | --- |
| Auditory naming LI |  | 𝛽= -0.453, p = 0.063, 95CI= -0.782:-0.144 |
| Auditory Naming fMRI LI |  | 𝛽= 0.208, p = 0.364, 95CI= -0.103:0.531 |
| Preoperative Scores |  | 𝛽= -0.244, p = 0.117, 95CI= -0.456:-0.042 |
| Gender |  | 𝛽= -0.84, p = 0.084, 95CI= -1.466:-0.192 |
| Operation Type |  | 𝛽= -1.813, p = 0.021, 95CI= -2.76:-0.853 |
| Epilepsy Duration |  | 𝛽= 0.36, p = 0.752, 95CI= -1.222:1.964 |
| Age of Onset |  | 𝛽= 0.349, p = 0.776, 95CI= -1.357:2.089 |
| Education |  | 𝛽= -0.136, p = 0.605, 95CI= -0.507:0.229 |
| Age |  | 𝛽= -0.04, p = 0.686, 95CI= -0.178:0.096 |
| Time |  | 𝛽= 0.128, p = 0.374, 95CI= -0.134:0.433 |
| Patient random effects |  | Variance=0.434, SD=0.659 |
| Overall Model Performance |  | X^2^(1)=7.319, p=0.007, Marginal R^2^=0.51 |

| **Picture naming Lateralisation Index & change in naming Z-scores** |  | **Statistics** |
| --- | --- | --- |
| Picture naming LI |  | 𝛽= -0.496, p = 0.051, 95CI= -0.821:-0.168 |
| Picture Naming fMRI LI |  | 𝛽= -0.166, p = 0.599, 95CI= -0.61:0.277 |
| Preoperative Scores |  | 𝛽= -0.144, p = 0.308, 95CI= -0.339:0.051 |
| Gender |  | 𝛽= -1.024, p = 0.139, 95CI= -1.964:-0.093 |
| Operation Type |  | 𝛽= -1.618, p = 0.039, 95CI= -2.609:-0.623 |
| Epilepsy Duration |  | 𝛽= -0.432, p = 0.733, 95CI= -2.214:1.362 |
| Age of Onset |  | 𝛽= -0.618, p = 0.657, 95CI= -2.579:1.355 |
| Education |  | 𝛽= -0.182, p = 0.513, 95CI= -0.573:0.208 |
| Age |  | 𝛽= 0.032, p = 0.772, 95CI= -0.125:0.188 |
| Time |  | 𝛽= 0.113, p = 0.428, 95CI= -0.16:0.391 |
| Patient random effects |  | Variance=0.412, SD=0.642 |
| Overall Model Performance |  | X^2^(1)=7.936, p=0.005, Marginal R^2^=0.53 |

Online resource 9. Linear mixed effect model results for group activation tract volumes and tract laterality indices with naming score change from preoperative to 4-month and 12-month postoperatively, accounting for preoperative language fMRI LI, preoperative naming scores, age, age at onset of seizures, epilepsy duration, surgery type, birth sex, and education status.

| **Auditory naming Tract volume & change in raw naming scores** |  | **Statistics** |
| --- | --- | --- |
| Auditory naming Group Activation Tract volume |  | 𝛽= -0.306, p = 0.753, 95CI= -1.68:1.069 |
| Auditory Naming fMRI LI |  | 𝛽= 0.289, p = 0.779, 95CI= -1.167:1.744 |
| Preoperative Scores |  | 𝛽= -0.094, p = 0.649, 95CI= -0.387:0.199 |
| Gender |  | 𝛽= -4.223, p = 0.06, 95CI= -7.141:-1.334 |
| Operation Type |  | 𝛽= -5.023, p = 0.135, 95CI= -9.526:-0.529 |
| Epilepsy Duration |  | 𝛽= -1.691, p = 0.754, 95CI= -9.312:5.92 |
| Age of Onset |  | 𝛽= -2.276, p = 0.692, 95CI= -10.393:5.821 |
| Education |  | 𝛽= 0.016, p = 0.99, 95CI= -1.724:1.745 |
| Age |  | 𝛽= 0.205, p = 0.649, 95CI= -0.429:0.84 |
| Time |  | 𝛽= 0.432, p = 0.378, 95CI= -0.536:1.37 |
| Patient random effects |  | Variance=11.23, SD=3.351 |
| Overall Model Performance |  | X^2^(1)=0.208, p=0.648, Marginal R^2^=0.32 |

| **Picture naming Tract volume & change in raw naming scores** |  | **Statistics** |
| --- | --- | --- |
| Picture Naming Group Activation Tract Volume |  | 𝛽= -0.612, p = 0.591, 95CI= -2.213:0.998 |
| Picture Naming fMRI LI |  | 𝛽= -0.682, p = 0.658, 95CI= -2.866:1.487 |
| Preoperative Scores |  | 𝛽= -0.089, p = 0.652, 95CI= -0.365:0.189 |
| Gender |  | 𝛽= -4.947, p = 0.137, 95CI= -9.445:-0.511 |
| Operation Type |  | 𝛽= -4.763, p = 0.207, 95CI= -9.926:0.349 |
| Epilepsy Duration |  | 𝛽= -1.091, p = 0.869, 95CI= -10.475:8.326 |
| Age of Onset |  | 𝛽= -1.646, p = 0.819, 95CI= -11.836:8.574 |
| Education |  | 𝛽= 0.116, p = 0.927, 95CI= -1.679:1.906 |
| Age |  | 𝛽= 0.147, p = 0.8, 95CI= -0.678:0.968 |
| Time |  | 𝛽= 0.424, p = 0.386, 95CI= -0.553:1.354 |
| Patient random effects |  | Variance=10.872, SD=3.297 |
| Overall Model Performance |  | X^2^(1)=0.592, p = 0.441, Marginal R^2^=0.33 |

| **Auditory naming Lateralisation Index & change in raw naming scores** |  | **Statistics** |
| --- | --- | --- |
| Auditory naming Group Activation Tract LI |  | 𝛽= -0.161, p = 0.9, 95CI= -1.969:1.639 |
| Auditory Naming fMRI LI |  | 𝛽= 0.373, p = 0.708, 95CI= -1.037:1.779 |
| Preoperative Scores |  | 𝛽= -0.087, p = 0.703, 95CI= -0.408:0.234 |
| Gender |  | 𝛽= -4.327, p = 0.065, 95CI= -7.381:-1.307 |
| Operation Type |  | 𝛽= -5.142, p = 0.143, 95CI= -9.857:-0.443 |
| Epilepsy Duration |  | 𝛽= -1.541, p = 0.775, 95CI= -9.166:6.072 |
| Age of Onset |  | 𝛽= -2.13, p = 0.712, 95CI= -10.285:6.006 |
| Education |  | 𝛽= 0.13, p = 0.924, 95CI= -1.811:2.064 |
| Age |  | 𝛽= 0.188, p = 0.682, 95CI= -0.46:0.836 |
| Time |  | 𝛽= 0.43, p = 0.38, 95CI= -0.54:1.367 |
| Patient random effects |  | Variance=11.333, SD=3.367 |
| Overall Model Performance |  | X^2^(1)=0.036, p=0.849, Marginal R^2^=0.32 |

| **Picture naming Lateralisation Index & change in raw naming scores** |  | **Statistics** |
| --- | --- | --- |
| Picture naming Group Activation Tract LI |  | 𝛽= 0.96, p = 0.364, 95CI= -0.504:2.425 |
| Picture Naming fMRI LI |  | 𝛽= -0.832, p = 0.578, 95CI= -2.94:1.259 |
| Preoperative Scores |  | 𝛽= -0.017, p = 0.929, 95CI= -0.292:0.258 |
| Gender |  | 𝛽= -4.827, p = 0.134, 95CI= -9.164:-0.557 |
| Operation Type |  | 𝛽= -5.544, p = 0.115, 95CI= -10.241:-0.886 |
| Epilepsy Duration |  | 𝛽= 3.171, p = 0.615, 95CI= -5.69:12.046 |
| Age of Onset |  | 𝛽= 3.201, p = 0.648, 95CI= -6.657:13.066 |
| Education |  | 𝛽= 0.146, p = 0.904, 95CI= -1.576:1.859 |
| Age |  | 𝛽= -0.214, p = 0.696, 95CI= -0.989:0.558 |
| Time |  | 𝛽= 0.425, p = 0.385, 95CI= -0.55:1.358 |
| Patient random effects |  | Variance=10.209, SD=3.195 |
| Overall Model Performance |  | X^2^(1)=1.744, p=0.187, Marginal R^2^=0.35 |

| **Auditory naming Tract volume & change in naming Z-scores** |  | **Statistics** |
| --- | --- | --- |
| Auditory naming Group Level Tract volume |  | 𝛽= -0.137, p = 0.574, 95CI= -0.478:0.205 |
| Auditory Naming fMRI LI |  | 𝛽= -0.045, p = 0.862, 95CI= -0.412:0.323 |
| Preoperative Z-scores |  | 𝛽= -0.21, p = 0.262, 95CI= -0.467:0.047 |
| Gender |  | 𝛽= -1.086, p = 0.053, 95CI= -1.813:-0.368 |
| Operation Type |  | 𝛽= -1.437, p = 0.086, 95CI= -2.53:-0.339 |
| Epilepsy Duration |  | 𝛽= 0.048, p = 0.971, 95CI= -1.842:1.936 |
| Age of Onset |  | 𝛽= -0.047, p = 0.974, 95CI= -2.078:1.979 |
| Education |  | 𝛽= -0.02, p = 0.948, 95CI= -0.449:0.406 |
| Age |  | 𝛽= 0.0004, p = 0.997, 95CI= -0.161:0.16 |
| Time |  | 𝛽= 0.109, p = 0.442, 95CI= -0.168:0.388 |
| Patient random effects |  | Variance=0.647, SD=0.804 |
| Overall Model Performance |  | X^2^(1)=0.670, p=0.413, Marginal R^2^=0.39 |

| **Picture naming Tract volume & change in naming Z-scores** |  | **Statistics** |
| --- | --- | --- |
| Picture naming Group Level Tract volume |  | 𝛽= -0.191, p = 0.494, 95CI= -0.582:0.204 |
| Picture Naming fMRI LI |  | 𝛽= -0.136, p = 0.718, 95CI= -0.67:0.393 |
| Preoperative Scores |  | 𝛽= -0.19, p = 0.268, 95CI= -0.425:0.046 |
| Gender |  | 𝛽= -1.25, p = 0.128, 95CI= -2.361:-0.158 |
| Operation Type |  | 𝛽= -1.4, p = 0.136, 95CI= -2.663:-0.148 |
| Epilepsy Duration |  | 𝛽= -0.11, p = 0.946, 95CI= -2.398:2.198 |
| Age of Onset |  | 𝛽= -0.22, p = 0.901, 95CI= -2.711:2.291 |
| Education |  | 𝛽= -0.003, p = 0.991, 95CI= -0.445:0.437 |
| Age |  | 𝛽= 0.013, p = 0.929, 95CI= -0.19:0.214 |
| Time |  | 𝛽= 0.107, p = 0.451, 95CI= -0.172:0.382 |
| Patient random effects |  | Variance=0.622, SD=0.788 |
| Overall Model Performance |  | X^2^(1)=0.941, p = 0.332, Marginal R^2^=0.40 |

| **Auditory naming Lateralisation Index & change in naming Z-scores** |  | **Statistics** |
| --- | --- | --- |
| Auditory naming Group Activation Tract LI |  | 𝛽= -0.149, p = 0.637, 95CI= -0.593:0.294 |
| Auditory Naming fMRI LI |  | 𝛽= -0.022, p = 0.929, 95CI= -0.378:0.333 |
| Preoperative Scores |  | 𝛽= -0.219, p = 0.28, 95CI= -0.498:0.059 |
| Gender |  | 𝛽= -1.167, p = 0.045, 95CI= -1.914:-0.43 |
| Operation Type |  | 𝛽= -1.543, p = 0.078, 95CI= -2.684:-0.4 |
| Epilepsy Duration |  | 𝛽= 0.117, p = 0.93, 95CI= -1.782:2.014 |
| Age of Onset |  | 𝛽= 0.041, p = 0.977, 95CI= -2.014:2.091 |
| Education |  | 𝛽= 0.068, p = 0.838, 95CI= -0.405:0.539 |
| Age |  | 𝛽= -0.013, p = 0.909, 95CI= -0.18:0.153 |
| Time |  | 𝛽= 0.108, p = 0.449, 95CI= -0.172:0.384 |
| Patient random effects |  | Variance=0.654, SD=0.809 |
| Overall Model Performance |  | X^2^(1)=0.479, p=0.489, Marginal R^2^=0.39 |

| **Picture naming Lateralisation Index & change in naming Z-scores** |  | **Statistics** |
| --- | --- | --- |
| Picture naming Group Activation Tract LI |  | 𝛽= 0.187, p = 0.486, 95CI= -0.188:0.563 |
| Picture Naming fMRI LI |  | 𝛽= -0.176, p = 0.638, 95CI= -0.704:0.347 |
| Preoperative Scores |  | 𝛽= -0.125, p = 0.477, 95CI= -0.37:0.121 |
| Gender |  | 𝛽= -1.284, p = 0.115, 95CI= -2.376:-0.211 |
| Operation Type |  | 𝛽= -1.622, p = 0.073, 95CI= -2.799:-0.449 |
| Epilepsy Duration |  | 𝛽= 0.892, p = 0.573, 95CI= -1.324:3.117 |
| Age of Onset |  | 𝛽= 0.896, p = 0.611, 95CI= -1.571:3.369 |
| Education |  | 𝛽= 0.023, p = 0.939, 95CI= -0.41:0.454 |
| Age |  | 𝛽= -0.071, p = 0.61, 95CI= -0.265:0.123 |
| Time |  | 𝛽= 0.108, p = 0.45, 95CI= -0.173:0.384 |
| Patient random effects |  | Variance=0.619, SD=0.797 |
| Overall Model Performance |  | X^2^(1)=1.031, p=0.310, Marginal R^2^=0.41 |
